# Evaluating associations between the benefits and risks of drug therapy in type 2 diabetes: a joint modeling approach

**DOI:** 10.2147/CLEP.S179555

**Published:** 2018-12-14

**Authors:** John M Dennis, Beverley M Shields, Angus G Jones, Ewan R Pearson, Andrew T Hattersley, William E Henley, On behalf of the MASTERMIND consortium

**Affiliations:** 1 Health Statistics Group, University of Exeter Medical School, Exeter, UK, W.E.Henley@exeter.ac.uk; 2 National Institute for Health Research Exeter Clinical Research Facility, University of Exeter Medical School, Exeter, UK; 3 Division of Molecular and Clinical Medicine, Ninewells Hospital and Medical School, University of Dundee, Dundee, UK

**Keywords:** diabetes mellitus, type 2, drug-related side effects, HbA1c, hypoglycemia, joint model, precision medicine, thiazolidinediones, metformin, sulfonylurea compounds, ADOPT, edema

## Abstract

**Objective:**

Precision medicine drug therapy seeks to maximize efficacy and minimize harm for individual patients. This will be difficult if drug response and side effects are positively associated, meaning that patients likely to respond best are at increased risk of side effects. We applied joint longitudinal–survival models to evaluate associations between drug response (longitudinal outcome) and the risk of side effects (survival outcome) for patients initiating type 2 diabetes therapy.

**Study design and setting:**

Participants were randomized to metformin (MFN), sulfonylurea (SU), or thiazolidinedione (TZD) therapy in the A Diabetes Outcome Progression Trial (ADOPT) drug efficacy trial (n=4,351). Joint models were parameterized for 1) current HbA1c response (change from baseline in HbA1c) and 2) cumulative HbA1c response (total HbA1c change).

**Results:**

With MFN, greater HbA1c response did not increase the risk of gastrointestinal events (HR per 1% absolute greater current response 0.82 [95% CI 0.67, 1.01]; HR per 1% higher cumulative response 0.90 [95% CI 0.81, 1.00]). With SU, greater current response was associated with an increased risk of hypoglycemia (HR 1.41 [95% CI 1.04, 1.91]). With TZD, greater response was associated with an increased risk of edema (current HR 1.45 [95% CI 1.05, 2.01]; cumulative 1.22 [95% CI 1.07, 1.38]) but not fracture.

**Conclusion:**

Joint modeling provides a useful framework to evaluate the association between response to a drug and the risk of developing side effects. There may be great potential for widespread application of joint modeling to evaluate the risks and benefits of both new and established medications.

## Plain language summary

**Purpose of study:** An overlooked question in precision/stratified medicine and when evaluating new medications is: are the benefits and risks of a drug associated? Joint longitudinal-survival models can be applied to answer this question when, as in type 2 diabetes, drug response is measured by a longitudinal biomarker (HbA1c) and risks of side-effects can be represented as a time-to-event outcome.

**What did we do and find?** We used joint longitudinal–survival models to show novel associations between the benefit of greater drug response and the risk of common side effects for three glucose-lowering medications for patients with type 2 diabetes. Greater drug response was associated with an increased risk of hypoglycemia with sulfonylureas and edema with thiazolidinediones. In contrast, there was no evidence of an increased risk of gastrointestinal side effects with metformin.

**What do the findings mean?** Joint models provide a novel, flexible, and robust approach to study the associations between the risks and benefits of drug therapy. Precision/stratified medicine studies seeking to identify patients or subgroups likely to respond well to a drug should also evaluate whether the same patients are at increased risk of side effects.

## Introduction

There is an increasing interest in applying a precision medicine approach to select the most appropriate drug for a patient or subgroup of patients, in order to either improve response or reduce side effects.^1^,^2^ An important but overlooked question, particularly if side effects are a result of the primary pharmacological effect of the drug, is whether the patients most likely to benefit are also at greatest risk of side effects. Type 2 diabetes is an ideal candidate for precision medicine, as there are many drug options to lower blood glucose (as measured by HbA1c), but each drug has a different mechanism of action and specific side effects. However, the association between HbA1c response and side effects is unknown for all drug options. If patients likely to have a greater HbA1c response to a specific drug are also at increased risk of side effects, this may limit the clinical utility of any precision approach to type 2 diabetes therapy.

To date, no robust framework has been proposed to evaluate the association between drug response and risk of side effects. In type 2 diabetes, HbA1c is measured repeatedly over time (a longitudinal process), while side effect risk can be modeled as a time-to-event process. In this scenario, joint longitudinal–survival modeling is the preferred approach to evaluate the association between both processes.^3^–^6^ Joint models attempt to capture the true, unobserved, longitudinal trajectory (in reality, HbA1c is measured intermittently and is subjected to measurement error from random noise and biological variation). This means that joint models can reduce bias and improve efficiency compared with simpler approaches.^5^,^7^ Joint models have been applied in many diseases including recently in type 1 diabetes (autoantibodies and time to disease onset),^8^–^11^ but not to our knowledge in type 2 diabetes, or more broadly to evaluate the association between drug response and the risk of side effects.

In this study, we applied joint modeling to evaluate the association between drug response and the risk of established side effects for three widely used type 2 diabetes drugs and, thus, further evaluate the potential for precision drug therapy in type 2 diabetes.

## Methods

### Overview

Our aim was to understand whether the degree of glycemic response to three common glucose-lowering drugs altered the risk of developing a side effect. To answer this question, we examined the association between the following two outcomes: 1) HbA1c response (as measured by change from baseline in HbA1c) and 2) risk of developing a side effect (gastrointestinal [GI] events, hypoglycemia, edema, and fracture).

### Setting and design

We used individual participant level data from A Diabetes Outcome Progressing Trial (ADOPT) randomized trial,^12^ accessed through the Clinical Trial Data Transparency Portal under approval from GlaxoSmithKline (GSK) (Proposal-930).^13^ ADOPT was a prospective head-to-head drug trial including treatment-naive participants with type 2 diabetes who were randomized to metformin (MFN), the sulfonylurea (SU) glyburide, or the thiazolidinedione (TZD) rosiglitazone (n=4,351 participants). The aim of ADOPT was to evaluate the long-term efficacy of TZD therapy compared to SU and MFN, and the primary outcome was time to therapy failure (confirmed fasting plasma glucose ≥180 mg/dL). Study visits were every 2 months in year 1, then every 3 months up to 5 years. Clinically determined adverse events were recorded at each study visit, including GI events, hypoglycemia, edema, and fracture. Biomarkers including HbA1c were recorded at each visit. ADOPT participants in the intention to treat population with a valid baseline HbA1c were eligible for our study. Participants were censored if they reached the trial primary endpoint of glycemic failure, trial-recorded study withdrawal, or at 5 years after starting therapy as in the ADOPT main analysis.

### Study outcomes

Our time-to-event outcomes were the first occurrence of four established drug-specific side effects, over a 5-year period. For MFN, the outcome of interest was a GI event, for SU, it was hypoglycemia (patient self-reported), and for TZD, we evaluated edema and bone fractures.^12^ Each drug and side effect combination was analyzed separately. We excluded patients with a pre-trial history of edema from the edema analysis (6% of patients), but pre-trial hypoglycemia, GI, and fracture records were not available to do the same for other side effects. Due to the high number of GI events, we repeated the GI analysis restricted to only moderate/severe and severe events as sensitivity analysis. The longitudinal outcome of interest was HbA1c response as measured by change from baseline in HbA1c (HbA1c at each study visit [%] – baseline HbA1c [%]). Throughout HbA1c percentages refer to absolute values rather than percentage changes. To test the specificity of our findings, we repeated the analysis for each side effect for the other drugs.

### Statistical analysis

**Figure 1:**
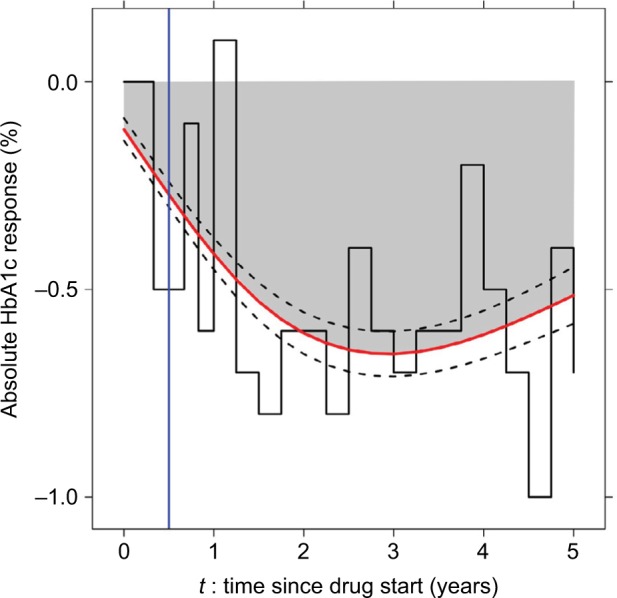
Approaches to estimating HbA1c (%) response. **Notes:** Model 1: estimate current HbA1c response using a joint model (red line with black dotted 95% CIs). Model 2: estimate cumulative HbA1c response using a joint model (gray-shaded area). Model 3: carry forward the most recently observed value of HbA1c response until the next measurement (LOCF approach, black step function). Model 4: take the observed HbA1c response at a single time point of 6 months (blue line). **Abbreviation:** LOCF, last observation carried forward.

We used a joint model with two parameterizations (Models 1 and 2) and two standard time-to-event models (Models 3 and 4), for comparison, to evaluate the association between HbA1c response and the risk of developing a side effect. A fundamental difference between each model was in the method to estimate HbA1c response, as illustrated in Figure 1. Each side effect was evaluated separately, and the same modeling approach was applied for each side effect. Participants were followed up for up to 5 years from randomization. As we were assessing the association between side effects and response, all participants required at least one pre-side effect HbA1c measure (meaning that participants with very early side effects were excluded: 4% of participants with edema, 3% of participants with fracture, 20% of participants with hypoglycemia, and 12% of participants with GI events). All models were adjusted for baseline HbA1c.^14^ Model setups were as follows.

#### Joint longitudinal–survival models

We used a maximum likelihood joint longitudinal–survival model to simultaneously assess the association between HbA1c response (longitudinal process) and the risk of developing a side effect (survival process). The joint model consisted of the following two parts: a longitudinal submodel and a survival sub-model linked through shared subject-specific random effects.^6^

In the general survival submodel, the hazard for patient *i* (*h_i_*(*t*)) can be represented as

$$h_{i}(t)=h_{0}(t)\exp(w_{i}^{T}\gamma +\alpha m_{i}(t))$$

where *h_0_* (*t*) is the baseline hazard, *w_i_* are baseline covariates, γ are regression coefficients, *m_i_*(*t*) is the “true, unobserved” longitudinal biomarker (estimated from the longitudinal submodel), and α quantifies the association between the longitudinal biomarker and the time-to-event process.^6^

We derived *m_i_*(*t*) from the observed HbA1c response data using a linear mixed effects model with a nonlinear term for time (as HbA1c response is typically nonlinear):

$$y_{i}(t)=m_{i}(t)+\varepsilon_{i}(t)$$

$$=\beta_{0}+\beta_{1}N{\left(t_{i}\right)}_{1}+\beta_{2}N{\left(t_{i}\right)}_{2}+\beta_{3}\;\text{Baseline HbA}1c+b_{i}{\;}_{0}+b_{i}{\;}_{1}N{\left(t_{i}\right)}_{1}+b_{i}{\;}_{2}N{\left(t_{i}\right)}_{2}+\varepsilon_{i}(t)$$

where *y_i_* is the observed HbA1c change from baseline and *m_i_* is the “true”, unobserved HbA1c change from baseline. *N*(*t_i_*)_1_ and *N*(*t_i_*)_2_ denote the basis for a nonlinear natural cubic spline of time with one internal knot at the 50th percentile of follow-up time (included in both the fixed and random effect parts of the longitudinal HbA1c submodel), *b_i_* is a vector of subject-specific random effects, *b_i_~ N* (O, $\tilde{D}$) where $\tilde{D}$ is the unstructured covariance matrix of random effects, ε*_i_* is the vector of residuals, and ε*_i_* ~ *N*(O,*σ*^2^), where *σ* 2 is the covariance matrix of the residuals.^6^ For models of hypoglycemia with MFN and edema with SUs, we used a linear term for the random effect of time to achieve model convergence.

*Model 1: joint model current value (JMcv).* To assess the association between the current value of HbA1c response and the risk of side effects (the standard formulation of the joint model), we incorporated *m_i_* from the longitudinal submodel as a time-dependent covariate in the survival submodel:

$$h_{i}(t)=h_{0}(t)\exp\{\gamma_{0}\text{Baseline}\;\text{HbA}1c+\alpha m_{i}(t)\}$$

*Model 2: joint model cumulative HbA1c (JMcum*). To evaluate whether the risk of side effects was associated with total rather than current HbA1c response, we specified a second formulation of the joint model to assess the association between cumulative HbA1c response (total HbA1c response estimated as area under the curve) and the risk of side effects, by including $\int_{0}^{t}m_{i}(s)ds$, the integral of the longitudinal HbA1c response trajectory up to time *t*, in the time-to-event submodel:^6^,^15^

$$h_{i}(t)=h_{0}(t)\exp\{\gamma_{0}\text{Baseline}\;\text{HbA}1c+\alpha \int_{0}^{t}m_{i}(s)ds\}$$

For Models 1 and 2, we used a B-spline with five internal knots to flexibly model the baseline hazard function. We examined the fit of submodels using residual plots. Models 1 and 2 were fitted using the JM package in R.^16^

*Model 3: last observation carried forward (LOCF*) *analysis*. We included observed HbA1c response (HbA1c at time *t* – baseline HbA1c) as a time-dependent covariate in a Cox proportional hazards model. This approach does not correct for measurement error and assumes that HbA1c response is constant between measurements. HRs represent the increased risk of a side effect for a 1-unit (%) absolute increase in the most recent value of HbA1c change from baseline at time *t*.

*Model 4: single estimate of HbA1c response at 6 months (6mR)*. We evaluated the association between HbA1c response at 6 months and the subsequent risk of developing a side-effect. In this two-stage approach, we first estimated a single estimate of HbA1c response as a change score at 6 months. In the second stage, we used this estimate as the exposure in a Cox hazards survival model with delayed entry to 6 months. Participants who developed a side effect prior to 6 months or had no HbA1c record at 6 months were excluded from this analysis.

## Results

The most common side effects were GI side effects with MFN (37%), followed by hypoglycemia with SU therapy (26%). TZD side effects were less common (edema 13% and fracture 7%; Table 1). The median follow-up was greater than 2.5 years in each cohort (for other participant characteristics, refer Table S1). Each side effect occurred more frequently on these therapies than on the comparator drugs (Table S2).

### Joint model associations between HbA1c response and risk of side effects

#### GI events

**Figure 2:**
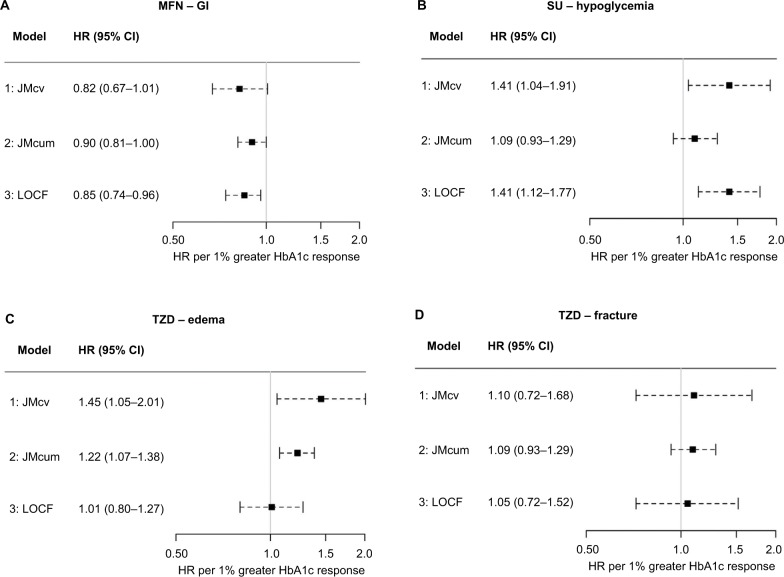
HRs for the association between HbA1c response and the risk of a drug-specific side effect (models 1–3). **Notes:** HRs (95% CI) represent the increase in the risk of side effect for a 1% greater absolute HbA1c response. A HR of greater than 1 indicates an increased risk of side effect with greater HbA1c response. **Abbreviations:** JMcum, joint model cumulative HbA1c; JMcv, joint model current value; LOCF, last observation carried forward; MFN, metformin; SU, sulfonylurea; TZD, thiazolidinedione.

With MFN, we found consistent evidence for an association between greater HbA1c response and reduced risk of a GI side effect (Figure 2A). We observed a similar association for moderate/severe GI events (20% of patients) and no association for severe GI events (3% of patients) (Table S3). We found no evidence of an association with TZDs and SUs (Tables 2 and S3).

#### Hypoglycemia

With SUs, we found that greater current HbA1c response was associated with an increased risk of hypoglycemia (Model 1: JMcv; Figure 2B). We found no evidence for an association between the risk of hypoglycemia and cumulative HbA1c response (Model 2: JMcum). With TZD therapy, although the absolute risk of hypoglycemia was much lower than with SU therapy (8 vs 26%), greater current and cumulative HbA1c responses were associated with an increased risk of hypoglycemia. There was no evidence of an association between response and hypoglycemia with MFN (Table 2).

#### Edema

With TZDs, greater current (Model 1: JMcv) and cumulative (Model 2: JMcum) HbA1c responses were associated with an increased risk of edema (Figure 2C). We found no evidence of an association between HbA1c response and the risk of edema with MFN and SUs (Table 2).

#### Fracture

With TZDs, we found no evidence for an association between HbA1c response and the risk of a fracture (Figure 2D). There was also no evidence of an association with MFN and SUs (Tables 2).

### Associations using standard time-to-event approaches

Results using the LOCF approach (Model 3: LOCF) were generally consistent with those from the current value joint models (Model 1: JMcv) (Table 2 and Figure 2). The exception was for TZDs and edema, for which, in contrast to the joint model, we found no evidence of an association using the LOCF model. Using Model 4: 6mR (where HbA1c response was estimated from a single 6 month value), we found no evidence of any association between HbA1c response and the risk of side effects except for GI events with MFN (HR per 1% absolute increase in 6-month HbA1c response, 0.74 [95% CI 0.60, 0.91], Table S5).

## Discussion

Our study shows that joint modeling can be a useful approach for evaluating associations between the benefits and risks of drug therapy. Using joint models for longitudinal and time-to-event data, we were able to show important differences in the associations between drug response and the risk of established side effects for three widely used type 2 diabetes drugs. We also found differences in the association between each of current and cumulative drug response and the risk of side-effects, suggesting underlying differences in the nature of associations for different drugs. Our results have implications for any precision medicine approach to type 2 diabetes therapy. More generally, they highlight the potential for the widespread application of joint longitudinal–survival modeling to evaluate the benefits and risks of both new and established medications.

### Advantages and disadvantages of joint models to evaluate the association between drug response and risk of side effects

We found a key advantage of joint models to be their flexibility. Different specifications of the joint model gave important additional insight into the underlying nature of associations between HbA1c response and side effects. These insights fitted with what is known about the pharmacological action of the different drugs. Current, but not cumulative, HbA1c response was associated with an increased risk of hypoglycemia with SUs. This is expected as hypoglycemia is a side effect related to short-term fluctuations in blood glucose, rather than long-term exposure. In contrast, for edema with TZDs, which is less likely to relate to short-term fluctuations in blood glucose, we observed associations for both current and cumulative HbA1c responses.

We also found associations with joint models that were missed by simpler approaches. With edema with TZD therapy, there was no association using the LOCF approach but a clear association using both specifications of the joint model. This is likely due to the reduced bias and increased efficiency of the joint model compared with the LOCF approach, which does not correct for measurement error in the longitudinal HbA1c response.^5^,^7^ In general, HRs using the LOCF approach had the same direction of association but were attenuated compared with those obtained from the current value joint model, in keeping with previous comparisons.^4^,^17^ We found that a single measure of HbA1c at 6 months was insufficient to show the evidence of an association between HbA1c response and side effects, with the exception of GI side effects with MFN where the association was consistent with the joint model.

There are some settings where joint models may be more limited. ADOPT was a large randomized, double-blinded trial, and in this dataset, we found joint models to be useful to evaluate the association between response and relatively common side effects. Increasingly, similar trial datasets are available for researchers to address secondary research questions.^13^,^18^ It may be more challenging to apply joint modeling in other datasets. In particular, the potential of recording bias should be considered if conducting similar studies in electronic health records, although greater sample size may offer the opportunity to study rarer side effects. Testing the specificity of results to drugs known to cause the side effect by comparison with “negative control” drugs may be a useful starting point. Joint models may also be harder to apply to study associations between drug response and acute or allergic side effects that occur immediately after starting therapy. This was apparent in our analysis, as although we included over 1,000 participants for each drug, participants who developed an early side effect prior to a first on-therapy HbA1c were excluded, and this is a particular limitation of our analysis of hypoglycemia with SUs. Another limitation of the joint modeling framework applied in this study is the assumption of a fixed association between longitudinal HbA1c and the risk of each side effect. While inspection of residual plots indicated that this was an appropriate strategy, it is certainly plausible that associations could change with therapy duration, and incorporating duration of therapy as a time-varying effect within the joint modeling framework would be of considerable interest. Similarly, an extension of the joint modeling framework to robustly incorporate drug dose could yield further insight to complement the response:side effect associations evaluated in this study. Evaluating the impact of dose is a particular challenge in trials of drug efficacy such as ADOPT, as participants could be both uptitrated based on reaching glycemic thresholds and downtitrated if a randomized medication was poorly tolerated.

### Implications for a precision medicine approach to type 2 diabetes therapy

Our findings for the different drugs have implications for any future precision medicine approach to type 2 diabetes therapy. Greater MFN drug response was not associated with an increased risk of GI side effects, and this suggests great potential to target therapy if patients likely to have greater drug response can be robustly identified.^19^ However, targeting SUs and TZDs to patients may be difficult as good responders are likely to be at increased risk of, respectively, hypoglycemia and edema. Our findings highlight the vital importance of considering both differential drug response and the risk of side effects in precision medicine studies, and this has been overlooked in previous work.^20^,^21^

Our findings do not however preclude a precision medicine approach for SUs and TZDs. Identification of characteristics associated with either, but not both, improved drug response or lower risk of side effects may allow the targeting of these therapies. Furthermore, decisions on therapy should ultimately be informed by absolute rather than relative risks of benefit or harm.^1^ For example, if patients likely to respond well to a TZD can be identified, then, a TZD may still be an appropriate option for patients whose absolute risk of developing a side effect is sufficiently low.

### Comparison with other studies

To our knowledge, this is the first evaluation of the association between HbA1c response and the risk of side effects for any of the three drugs, except for hypoglycemia with SUs. Our results for SUs are consistent with previous observational studies that have examined the association between hypoglycemia and achieved on-therapy HbA1c (rather than HbA1c response).^22^,^23^ In the ACCORD trial, participants with the greatest HbA1c response at 4 months had a reduced rather than increased risk of hypoglycemia, although this can be explained by the fact that, in ACCORD, the participants with least initial response were more likely to be on insulin, the therapy with by far the strongest association with hypoglycemia.^24^

In this study, we found an unexpected association between greater response to TZD therapy and an increased risk of hypoglycemia, but no evidence of an association with MFN response, which would have indicated a positive association between increased drug response and increased risk of hypoglycemia was a more general characteristic of glucose-lowering therapy. This is an interesting finding for which there is no clear biological explanation, and it would be of interest to examine whether the association can be replicated in other datasets. The association between edema and HbA1c response with TZDs is not unexpected as the mechanisms underlying both glucose-lowering and fluid retention are thought to relate to Peroxisome proliferator-activated receptor gamma (PPAR-**γ**) stimulation.^25^ With MFN, there is no clear biological reason for the association between greater HbA1c response and a lower risk of GI events. One possible explanation is decreased drug adherence in patients experiencing mild GI symptoms prior to the event being recorded.

### Future work

There is great potential to apply joint modeling to evaluate the association between drug response and the risk of side effects for the other drug options in type 2 diabetes and to study drug therapy in other diseases. Our findings also suggest a potential application of joint modeling as an efficient tool for understanding the risk–benefit trade-off at the individual level in drug development.^26^ For precision medicine, the joint models used in this study could be extended to explore clinical features and biomarkers associated with drug response, the risk of side effects, or both.^27^,^28^ Alternative model specifications, such as evaluation of the effect of HbA1c response slope,^6^ the weighting of cumulative HbA1c effects by recency,^15^ the incorporation of multiple longitudinal biomarkers,^29^ and exploration of time-varying drug effects, may provide further insight into the nature of associations between response and side effects. Similarly, incorporation of robust dose adjustment within the joint modeling framework, for example, testing weighted cumulative drug associations,^30^,^31^ could allow much greater understanding of the impact of different levels of drug exposure on both response and adverse events. Many of these are areas of current methodological development; a general mathematical presentation of joint modeling for simultaneously evaluating risks and benefits of medication would be a useful next step.

## Conclusion

Joint modeling is a useful and efficient method to evaluate associations between continuous drug response and time to side effects. Our study suggests the potential for the application of joint modeling in both drug development and precision medicine research to evaluate the benefits and risks of medications. In type 2 diabetes, any future precision approach to SU and TZD therapy should consider the likely increased risk of, respectively, hypoglycemia and edema, if targeting these therapies at patients likely to have the greatest drug response.

## Data statement

No additional data are available from the authors, although the individual participant data from the ADOPT trial used in this study are available from GlaxoSmithKline on application via www.clinicalstudydatarequest.com.

## Supplementary Materials



## Figures and Tables

**Table 1:** Participant numbers and study follow-up for each primary drug: side effect cohort (Models 1–3)

	Metformin – GI	SU – hypo	TZD – edema	TZD – fracture
Number of participants	1,200	1,052	1,241	1,311
Number of events (%)	440 (37%)	270 (26%)	164 (13%)	88 (7%)
Baseline HbA1c (%)	7.3 (6.7; 7.9)	7.3 (6.7; 7.9)	7.3 (6.7; 7.9)	7.3 (6.7; 7.9)
Number of recorded HbA1c	13 (6; 19)	12 (5; 19)	18 (9; 20)	18 (10; 21)
Study follow-up (years)	2.8 (1.0; 4.2)	2.5 (0.9; 4.2)	4.0 (1.8; 4.7)	4.0 (2.1; 4.7)

**Note:** Data are median (IQR) unless stated (refer Table S4 for participants included in Model 4).

**Abbreviations:** GI, gastrointestinal; SU, sulfonylurea; TZD, thiazolidinedione.

**Table 2:** HRs for the association between HbA1c response and risk of side effects (models 1–3)

Side effect	Model 1: JMcv	Model 2: JMcum	Model 3: LOCF
MFN			
GI	0.82 (0.67, 1.01), *P*=0.06	0.90 (0.81, 1.00), *P*=0.06	0.85 (0.74, 0.96), *P*=0.01
Hypoglycemia	1.01 (0.63, 1.62), *P*=0.96	1.22 (0.93, 1.60), *P*=0.15	1.19 (0.88, 1.60), *P*=0.25
Edema	1.16 (0.70, 1.92), *P*=0.58	1.09 (0.88, 1.36), *P*=0.42	1.07 (0.74, 1.56), *P*=0.71
Fracture	0.83 (0.48, 1.44), *P*=0.51	1.00 (0.78, 1.27), *P*=0.98	0.98 (0.69, 1.39), *P*=0.92
SU			
GI	0.88 (0.69, 1.11), *P*=0.28	1.03 (0.92, 1.17), *P*=0.58	0.90 (0.77, 1.05), *P*=0.19
Hypoglycemia	1.41 (1.04, 1.91), *P*=0.03	1.09 (0.93, 1.29), *P*=0.28	1.41 (1.12, 1.77), *P*=0.003
Edema	1.31 (0.85, 2.02), *P*=0.23	1.09 (0.87, 1.36), *P*=0.45	0.87 (0.67, 1.13), *P*=0.28
Fracture	1.16 (0.70, 1.92), *P*=0.58	1.09 (0.88, 1.36), *P*=0.42	1.00 (0.64, 1.58), *P*=0.68
TZD			
GI	1.21 (0.94, 1.55), *P*=0.13	1.05 (0.93, 1.18), *P*=0.44	1.04 (0.87, 1.26), *P*=0.65
Hypoglycemia	1.98 (1.25, 3.15), *P*=0.004	1.37 (1.11, 1.7), *P*=0.003	1.44 (0.98, 2.12), *P*=0.07
Edema	1.45 (1.05, 2.01), *P*=0.03	1.22 (1.07, 1.38), *P*=0.003	1.01 (0.80, 1.27), *P*=0.94
Fracture	1.10 (0.72, 1.68), *P*=0.65	1.09 (0.93, 1.29), *P*=0.28	1.05 (0.72, 1.52), *P*=0.81

**Notes:** HRs (95% CI) represent the increase in risk of a side effect for a 1% greater absolute HbA1c response. A HR of greater than 1 indicates an increased risk of a side effect with greater HbA1c response.

**Abbreviations:** GI, gastrointestinal; JMcum, joint model cumulative HbA1c; JMcv, joint model current value; LOCF, last observation carried forward; MFN, metformin; SU, sulfonylurea; TZD, thiazolidinedione.

## Abbreviations

ADOPT: A Diabetes Outcome Progressing Trial
GSK: GlaxoSmithKline
MFN: metformin
SU: sulfonylurea
TZD: thiazolidinedione
