# Supplementary material for: Evaluating associations between the benefits and risks of drug therapy in type 2 diabetes: a joint modeling approach
Source: Clin Epidemiol. 2018 Dec 14;10:1869–77. doi: 10.2147/CLEP.S179555 (PMC6298877; doi:10.2147/CLEP.S179555)
Supplement: Supplementary file 1 [file CLEP-10-1869-179555.docx]

**Supplementary materials to:**

**Evaluating associations between the benefits and risks of drug therapy in type 2 diabetes: A joint modelling approach**

**Authors:** John M. Dennis, Beverley M. Shields, Angus G. Jones, Ewan R. Pearson, Andrew T. Hattersley, William E. Henley, on behalf of the MASTERMIND consortium

**Table S1: Participant baseline characteristics for all drug:side-effect cohorts (Models 1-3).** Data are mean (SD) unless stated.

|  | MFN:GI | SU:HYPO | TZD:OEDEMA | TZD:FRAC |
| --- | --- | --- | --- | --- |
| No. of participants | 1206 | 1052 | 1198 | 1311 |
| Baseline HbA1c (%) | 7.3 (0.9) | 7.3 (0.9) | 7.3 (0.9) | 7.3 (0.9) |
| Age at therapy (years) | 57 (10) | 57 (10) | 56 (10) | 56 (10) |
| Sex (% male) | 61% | 60% | 56% | 55% |
| Duration of diabetes (years) | 1.0 (1.0) | 1.0 (1.0) | 1.0 (0.9) | 1.0 (1.0) |
| BMI (kg/m2) | 32 (6) | 32 (6) | 31 (6) | 31 (6) |

**Table S2: Patient outcomes and study follow-up for all drug:side-effect cohorts (Models 1-3).**

|  | No. of participants | No. of events (%) | Mean (SD) study follow-up |
| --- | --- | --- | --- |
| Gastro-intestinal |  |  |  |
| **MFN** | **1200** | **440 (37%)** | **2.8 (1.7)** |
| SU | 1206 | 281 (23%) | 2.8 (1.7) |
| TZD | 1252 | 293 (23%) | 3.1 (1.6) |
| Hypoglycaemia |  |  |  |
| MFN | 1286 | 119 (9%) | 3.3 (1.6) |
| **SU** | **1052** | **270 (26%)** | **2.6 (1.7)** |
| TZD | 1281 | 104 (8%) | 3.3 (1.6) |
| Oedema |  |  |  |
| MFN | 1248 | 78 (6%) | 3.4 (1.6) |
| SU | 1198 | 91 (8%) | 3.5 (1.6) |
| **TZD** | **1241** | **164 (13%)** | **3.3 (1.6)** |
| Fracture |  |  |  |
| MFN | 1320 | 53 (4%) | 3.4 (1.6) |
| SU | 1320 | 53 (4%) | 3.4 (1.6) |
| **TZD** | **1311** | **88 (7%)** | **3.4 (1.6)** |

**Table S3: Hazard ratios for the association between HbA1c response and risk of gastrointestinal side-effects of all, moderate/severe and severe intensity (Models 1-3).** Hazard ratios (95% Confidence Intervals) represent the increase in risk of a GI side-effect for a 1% greater absolute HbA1c response**.** A hazard ratio greater than 1 indicates an increased risk of a GI side-effect with greater HbA1c response.

|  | Model 1: JMcv | Model 2: JMcum | Model 3: LOCF |  |
| --- | --- | --- | --- | --- |
|  | **MFN** | | |  |
| All | 0.82 (0.67*,* 1.01), *P=*0.06 | 0.90 (0.81*,* 1.00), *P=*0.06 | 0.85 (0.74*,* 0.96), *P=*0.01 |  |
| Moderate/Severe | 0.73 (0.56*,* 0.95), *P=*0.02 | 0.89 (0.77*,* 1.03), *P=*0.10 | 0.76 (0.65*,* 0.89), p<0.001 |  |
| Severe | 1.28 (0.60*,* 2.74), *P=*0.53 | 1.04 (0.74*,* 1.48), *P=*0.80 | 0.98 (0.69*,* 1.40), *P=*0.91 |  |
|  | **SU** | | | |
| All | 0.88 (0.69*,* 1.11), *P=*0.28 | 1.03 (0.92*,* 1.17), *P=*0.58 | 0.90 (0.77*,* 1.05), *P=*0.19 |  |
| Moderate/Severe | 0.85 (0.62*,* 1.17), *P=*0.32 | 0.91 (0.78*,* 1.07), *P=*0.25 | 0.91 (0.73*,* 1.14), *P=*0.43 |  |
| Severe | 1.07 (0.51*,* 2.22), *P=*0.87 | 0.96 (0.69*,* 1.34), *P=*0.81 | 0.87 (0.55*,* 1.36), *P=*0.54 |  |
|  | **TZD** | | |  |
| All | 1.21 (0.94*,* 1.55), *P=*0.13 | 1.05 (0.93*,* 1.18), *P=*0.44 | 1.04 (0.87*,* 1.26), *P=*0.65 |  |
| Moderate/Severe | 1.16 (0.75*,* 1.31), *P=*0.38 | 1.06 (0.90*,* 1.24), *P=*0.51 | 0.99 (0.75*,* 1.31), *P=*0.95 |  |
| Severe | 1.15 (0.53*,* 2.52), *P=*0.72 | 1.02 (0.73*,* 1.43), *P=*0.90 | 1.13 (0.67*,* 1.13), *P=*0.64 |  |

**Table S4: Number of participants and side effect events for Model 4: 6 month response.** Participants were included if they had a valid baseline HbA1c and a valid on-therapy HbA1c at 6 months, and had no record of the side effect of interest prior to the 6 month HbA1c.

| Side-effect | No. of patients (No. of events) | | |
| --- | --- | --- | --- |
|  | MFN | SU | TZD |
| Gastro-intestinal | 1025 (329) | 1057 (212) | 1114 (238) |
| Hypoglycaemia | 1149 (78) | 879 (162) | 1156 (79) |
| Oedema | 1145 (68) | 1094 (73) | 1129 (144) |
| Fracture | 1210 (38) | 1167 (34) | 1200 (80) |

**Table S5: Hazard ratios for the association between HbA1c response and risk of side-effects for Model 4: 6m Response.** Hazard ratios represent the increase in risk of a side-effect for a 1% greater absolute HbA1c response at 6 months**.** A hazard ratio greater than 1 indicates an increased risk of a side-effect with greater HbA1c response.

|  | Gastrointestinal | Hypoglycaemia | Oedema | Fracture |
| --- | --- | --- | --- | --- |
| **MFN** | **0.74 (0.60-0.91), p<0.01** | 1.21 (0.78-1.88), p=0.38 | 1.28 (2.11-2.93), p=1.29 | 0.82 (0.43-1.57), p=0.55 |
| **SU** | 0.86 (0.71-1.06), p=0.16 | **1.04 (0.79-1.35), p=0.79** | 0.94 (0.65-1.36), p=0.75 | 0.94 (0.55-1.63), p=0.84 |
| **TZD** | 1.24 (0.97-1.57), p=0.08 | 1.01 (0.67-1.53), p=0.95 | **1.06 (0.78-1.43), p=0.72** | **1.20 (0.79-1.80), p=0.39** |
